# Supplementary material for: Correlates of the HIV-associated neurocognitive disorders among adults living with HIV in Dodoma region, central Tanzania: A cross-sectional study
Source: PLoS One. 2023 May 25;18(5):e0285761. doi: 10.1371/journal.pone.0285761 (PMC10212098; doi:10.1371/journal.pone.0285761)
Supplement: Attachment — Submitted filename: Prevalence of HAND in Tanzania PLOS edits by reviewer of 9-3-22.pdf [file pone.0285761.s002.pdf]

PLOS ONE

Correlates of the HIV-associated neurocognitive disorders among adults living with HIV in Dodoma region, central Tanzania: a cross-sectional study

--Manuscript Draft--

**Manuscript Number:**	PONE-D-22-19422
**Article Type:**	Research Article
**Full Title:**	Correlates of the HIV-associated neurocognitive disorders among adults living with HIV in Dodoma region, central Tanzania: a cross-sectional study
**Short Title:**	Neurocognitive disorders in HIV
**Corresponding Author:**	Azan Nyundo, MD, Mmed<br>University of Dodoma College of Health and Allied Sciences: The University of Dodoma College of Health Sciences<br>Dodoma, Dodoma TANZANIA, UNITED REPUBLIC OF
**Keywords:**	HIV-associated neurocognitive disorders, Prevalence, Correlates
**Abstract:**	Background:<br>HIV-associated neurocognitive disorders (HAND) continue to manifest despite advancements and improved antiretroviral therapy coverage. Neurocognitive impairment is a significant predictor of poor prognosis related to poor antiretroviral therapy adherence and retention in HIV care.<br>Methods:<br>The study used a cross-sectional approach involving a sample of 397 participants attending care and treatment at Dodoma Regional Referral Hospital (DRRH) attained through systematic sampling. The combination of Montreal Cognitive Assessment (MoCA), International HIV Dementia Scale (IHDS), and The Lawton Instrumental Activity of Daily Living (IADL) were used to assess HIV-associated neurocognitive disorders. Factors associated with HAND were determined using univariate and multivariable logistic regression.<br>Results:<br>Of all 397 participants, 234(58.95%) met the criteria for HAND with 231(58.19%) having either Asymptomatic Neurocognitive Disorders (ANI) or Mild Neurocognitive Disorders (MND), and 3 (0.76%) had HIV dementia. Participants with HAND had significantly poorer performance in each cognitive domain on both MoCA and IHDS. Under multivariable regression, age of 55 years or above AOR: 3.493(95%CI: 1.052, 11.594), p=0.0410 and female gender were significantly associated with HAND while adherence to antiretroviral therapy AOR: 0.401(95%CI: 0.164, 0.977), p=0.0443, and attaining primary education AOR: 0.333(95%CI: 0.144, 0.770), p=0.0102 or secondary education AOR: 0.091(95%CI: 0.035, 0.238), p<0.0001 compared to having no formal education showed good cognitive performance.<br>Conclusion:<br>HIV-associated neurocognitive disorders are common in HIV, especially the less severe forms such as ANI and MND. Both socio-demographic and clinical variables may have significant influence neurocognitive functioning in the HIV population. The findings underscores the need to integrate screening for neurocognitive disorders to mitigate its adverse implication and improve overall clinical outcomes.
**Order of Authors:**	Azan Nyundo, MD, Mmed
**Additional Information:**	
**Question**	**Response**
**Financial Disclosure**<br><br>Enter a financial disclosure statement that describes the sources of funding for the work included in this submission. Review the submission guidelines for detailed requirements. View published research	Yes- The author A.A.N received funding for data collection process for the study from Fogarty International Center of the National Institutes of Health "https://www.fic.nih.gov/" under award number D43TW009775. The funders played no role in the study design, data collection and analysis, decision to publish, preparation of the manuscript or supporting for publication processing charges.

Powered by Editorial Manager® and ProduXion Manager® from Aries Systems Corporation

articles from *PLOS ONE* for specific examples.<br><br>This statement is required for submission and **will appear in the published article** if the submission is accepted. Please make sure it is accurate.<br><br>**Unfunded studies**<br>Enter: *The author(s) received no specific funding for this work.*<br><br>**Funded studies**<br>Enter a statement with the following details:<br>• Initials of the authors who received each award<br>• Grant numbers awarded to each author<br>• The full name of each funder<br>• URL of each funder website<br>• Did the sponsors or funders play any role in the study design, data collection and analysis, decision to publish, or preparation of the manuscript?<br>• **NO** - Include this sentence at the end of your statement: *The funders had no role in study design, data collection and analysis, decision to publish, or preparation of the manuscript.*<br>• **YES** - Specify the role(s) played.<br><br>* typeset	
**Competing Interests**<br><br>Use the instructions below to enter a competing interest statement for this submission. On behalf of all authors, disclose any competing interests that could be perceived to bias this work—acknowledging all financial support and any other relevant financial or non-financial competing interests.<br><br>This statement is required for submission and **will appear in the published article** if the submission is accepted. Please make sure it is accurate and that any funding sources listed in your Funding Information later in the submission form are also declared in your Financial Disclosure statement.	No-authors have no competing interests

View published research articles from *PLOS ONE* for specific examples.<br><br>**NO authors have competing interests**<br><br>Enter: *The authors have declared that no competing interests exist.*<br><br>**Authors with competing interests**<br><br>Enter competing interest details beginning with this statement:<br><br>*I have read the journal's policy and the authors of this manuscript have the following competing interests: [insert competing interests here]*<br><br>* typeset	
**Ethics Statement**<br><br>Enter an ethics statement for this submission. This statement is required if the study involved:<br><br>• Human participants<br>• Human specimens or tissue<br>• Vertebrate animals or cephalopods<br>• Vertebrate embryos or tissues<br>• Field research<br><br>Write "N/A" if the submission does not require an ethics statement.<br><br>General guidance is provided below. Consult the submission guidelines for detailed instructions. **Make sure that all information entered here is included in the Methods section of the manuscript.**	The study was approved by the local IRB of the Dodoma University Ethical and Research Committee with reference UDOM/ DRP/134/VOL V/91 once the proposal was fully established and merited approval. Trained research assistants who are medical doctors at the level of registrar provided participants with accurate, detailed information regarding the study. If a participant suffered from a neurological, psychiatric, or any other medical disorder needing urgent treatments, they were referred to specialized care based on the locally agreed protocols. Written informed consent was provided for those who could read and write; otherwise verbal informed consent was to be used as an alternative under the witness of one's choice. No minor was included in the study, if a participant could not consent due to medical or any other reason, a custodian who had to be a close relative was to provide assent for the patient to be involved in the study.

Powered by Editorial Manager® and ProduXion Manager® from Aries Systems Corporation

**Format for specific study types**<br><br>**Human Subject Research (involving human participants and/or tissue)**<br>• Give the name of the institutional review board or ethics committee that approved the study<br>• Include the approval number and/or a statement indicating approval of this research<br>• Indicate the form of consent obtained (written/oral) or the reason that consent was not obtained (e.g. the data were analyzed anonymously)<br><br>**Animal Research (involving vertebrate animals, embryos or tissues)**<br>• Provide the name of the Institutional Animal Care and Use Committee (IACUC) or other relevant ethics board that reviewed the study protocol, and indicate whether they approved this research or granted a formal waiver of ethical approval<br>• Include an approval number if one was obtained<br>• If the study involved *non-human primates*, add *additional details* about animal welfare and steps taken to ameliorate suffering<br>• If anesthesia, euthanasia, or any kind of animal sacrifice is part of the study, include briefly which substances and/or methods were applied<br><br>**Field Research**<br><br>Include the following details if this study involves the collection of plant, animal, or other materials from a natural setting:<br>• Field permit number<br>• Name of the institution or relevant body that granted permission	
**Data Availability**<br><br>Authors are required to make all data underlying the findings described fully available, without restriction, and from the time of publication. PLOS allows rare exceptions to address legal and ethical concerns. See the PLOS Data Policy and FAQ for detailed information.	Yes - all data are fully available without restriction

A Data Availability Statement describing where the data can be found is required at submission. Your answers to this question constitute the Data Availability Statement and **will be published in the article**, if accepted.<br><br>**Important:** Stating 'data available on request from the author' is not sufficient. If your data are only available upon request, select 'No' for the first question and explain your exceptional situation in the text box.<br><br>Do the authors confirm that all data underlying the findings described in their manuscript are fully available without restriction?	
**Describe where the data may be found in full sentences. If you are copying our sample text, replace any instances of XXX with the appropriate details.**<br><br>• If the data are **held or will be held in a public repository**, include URLs, accession numbers or DOIs. If this information will only be available after acceptance, indicate this by ticking the box below. For example: *All XXX files are available from the XXX database (accession number(s) XXX, XXX.*).<br>• If the data are all contained **within the manuscript and/or Supporting Information files**, enter the following: *All relevant data are within the manuscript and its Supporting Information files.*<br>• If neither of these applies but you are able to provide **details of access elsewhere**, with or without limitations, please do so. For example:<br><br>*Data cannot be shared publicly because of [XXX]. Data are available from the XXX Institutional Data Access / Ethics Committee (contact via XXX) for researchers who meet the criteria for access to confidential data.*<br><br>*The data underlying the results presented in the study are available from (include the name of the third party*	All relevant data are within the manuscript and its Supporting Information files.

and contact information or URL).<br>• This text is appropriate if the data are owned by a third party and authors do not have permission to share the data.<br><br>* typeset	
Additional data availability information:	Tick here if the URLs/accession numbers/DOIs will be available only after acceptance of the manuscript for publication so that we can ensure their inclusion before publication.; Tick here if your circumstances are not covered by the questions above and you need the journal's help to make your data available.

Powered by Editorial Manager® and ProduXion Manager® from Aries Systems Corporation

Manuscript

Click here to access/download;Manuscript;Manuscript HAND plos one.docx

Edits and comments by PLOS reviewer JAMc 9-3-22

Title:

Correlates of the HIV-associated neurocognitive disorders among adults living with HIV in Dodoma region, central Tanzania: a cross-sectional study

Authors

Azan A Nyundo

Department of Psychiatry and Mental Health, School of Medicine, The University of Dodoma, Dodoma, Tanzania

Correspondence

Azan A. Nyundo

P O Box 395 Dodoma, Tanzania

Email: azannaj@gmail.com, azan.nyundo@udom.ac.tz

Abstract

Background:

HIV-associated neurocognitive disorders (HAND) continue to manifest despite advancements and improved antiretroviral therapy coverage. Neurocognitive impairment is a significant predictor of poor prognosis related to poor antiretroviral therapy adherence and retention in HIV care.

Methods:

This cross-sectional study examined 397 participants ~~attending~~ cared for ~~and treatment~~ at Dodoma Regional Referral Hospital (DRRH) and selected by systematic sampling. The combination of Montreal Cognitive Assessnt (MoCA), International HIV Dementia Scale

(IHDS), and The Lawton Instrumental Activity of Daily Living (IADL) were used to assess HIV-associated neurocognitive disorders. Factors associated with HAND were determined using univariate and multivariable logistic regression.

Results:

Of 397 participants, 234(59.1%) met the criteria for HAND with 231(58.2%) comprising asymptomatic neurocognitive disorder (ANI) or mild neurocognitive disorders (MND), and 3 (0.76%) HIV- associated dementia (HAD) Participants with HAND had significantly poorer performance in each cognitive domain on both MoCA and IHDS. Multivariable regression analysis found, age of 55 years or above AOR: 3.5 (95%CI: 1.1, 11.6), p=0.041 and female gender **(AOR?)** were were associated with HAND In contrast, adherence to antiretroviral therapy AOR: 0.40(95%CI: 0.16, 01.0), p=0.044, and attaining primary education AOR: 0.33 (95%CI: 0.14, 0.77), p=0.010 or secondary education AOR: 0.091(95%CI: 0.035, 0.24), p<0.0001 compared to having no formal education were protective against diagnosis of HAND.

Conclusion:

HIV-associated neurocognitive disorders, especially ANI and MND, are common in HIV-infected Tanzanians. Both socio-demographic and clinical variables influence risk of neurocognitive impairment in this population. Screening for mild neurocognitive disorders may be indicated if effective treatment becomes available.

Keywords: HIV-associated neurocognitive disorders, Prevalence, Correlates

Introduction

HIV-associated neurocognitive disorders (HAND) is a neurological complication attributable to HIV in the central nervous system (CNS), manifesting with deficits in memory, concentration, attention, and motor skills(1,2). For clinical and research purposes, FRASCATI criteria are widely used to categorize HAND into Asymptomatic Neurocognitive Impairment (ANI), Mild Neurocognitive Disorders (MND), and a more severe form called HIV-associated disorders (HAD)(3,4).

Over three decades decades, the lifespan of people living with HIV (PLWH) has significantly improved with highly active antiretroviral therapy (HAART). Furthermore, incidence of severe HAD has been reduced by ART; however, nearly half the population continues to present with some form of cognitive impairment within the spectrum of HAND(5).

The brain is the second most affected organ in HIV infection after the lungs; the cascade of events related to the pathogenesis of cognitive impairment commences during the early phases of HIV infection. The virus enters the brain parenchyma (6,7) through the infected macrophages and lymphocytes or by the passage of cell-free virus and virus release from the infected endothelial [cells(8). The absence of overt neuropathologies that were common before the cART era suggests clinical presentation of HAND is attributed to functional alteration of neuronal connectivity as opposed to gross neuronal loss or encephalitis(9,10).

HAND remains highly prevalent in the cART era, albeit with less advanced stages of HIV infection or severe neurocognitive impairment. Currently, the less severe ANI and MND are observed even among patients with high CD4+ count and undetectable viral load as opposed to pre- ART era, where low CD4+ count, low weight, and anemia are the significant risks for HAND (11). Nadir CD4+ count, advanced age, hypertension, dyslipidemia hepatitis C (HCV) co-infection, type of ART used, and psychosocial factors including depression, anxiety, and stigma also remain risk factors for HAND (12–15).

This study examines with standardized quantitative methods the prevalence of and risk factors for HAND among adult patients on ART attending an HIV specialty care clinic in the Dodoma region of Tanzania.

Materials and Methods

Study design

This is a prospective, cross-sectional, single site study.

Study settings

Dodoma is the capital of Tanzania, located in the central part of the country, with about 410,956 people as per the 2012 census(16). The study was conducted in the Dodoma region's main referral hospital, "Dodoma Regional and Referral Hospital" (DRRH). Care and treatment clinic (CTC) services are also conducted

in the hospital five days a week, covering up to 100 patients a day coming mostly from Dodoma, and few come outside of Dodoma. Other services provided at the CTC include ART medications, HIV-related counseling services, routine investigations such as CD4+ count, viral load, general medical care, and medical referrals in case of need.

Study population and sampling

During the study period from March to June 2020, the CTC had enrolled 10,288 patients on ART, of whom 3,708 were available for participation. Using a Kish Leslie formula $n=Z^2p\ (1-p)\ /d2$ for a single proportion, a sample size (n) of 384 was calculated, P estimated at 0.5. Z=1.96, d=0.05. A final sample size of 397 was attained through a systematic sampling procedure whereby attendance was used to identify and direct every third participant for an interview after inclusion and exclusion criteria were applied.

Inclusion/exclusion criteria

We included all patients at least 18 years of age and being on ART for a minimum of six months. The participants must be able to provide informed consent with adequate vision, hearing, articulation, and without disability of any of the upper limbs for neurocognitive assessment. Those who could not read and write in English and Swahili were excluded from the study. We also excluded those with active CNS infection, known complications of previous CNS infection, neurological disorders, and an active phase of a psychotic episode.

Data collection, variables, and measurements

Outcome variable:

HIV-associated Neurocognitive disorders. The neurocognitive assessment was determined using the Swahili-translated Montreal Cognitive Assessment (MoCA) and International HIV dementia scale (IHDS).

A meta-analysis found that a cutoff of 23/30 on MoCA has better diagnostic accuracy across all domains than the routinely used 26/30 cutoff score, especially in a population with less education(17).

Acceptable reliability, a sensitivity of

70% and specificity of 60% for MCI and sensitivity of 72% and specificity for

dementia in the rural Dodoma older adult population (18). **Were results of this study used as a source of normative performance in this study?** The instrument also has a receiver operating characteristic curve with an area under the curve of

0.71; MoCA has been widely used worldwide among people with or without symptomatic HAND(19) and is considered a practical screening tool for cognitive assessment in HIV-population(20).

HIV-associated neurocognitive impairment (HAND) was assessed using the combination of the score of Montreal Cognitive Assessment (MoCA), the International HIV-associated dementia scale (IHDS), and The Lawton

No neurocognitive impairment

Based on MoCA score $\geq$ 23 or IHDS score $\geq$ 10 and IADL score of $\geq$ 8.

Asymptomatic neurocognitive impairment (ANI) or **Mild neurocognitive disorder (MND) is** considered positive if the patient scores < 23 in MoCA and a score of <10 on IHDS and IADL score of ≥ 8.

HIV- Associated Dementia (HAD)

The patient had to score < 23 on MoCA, a score of < 10 on IHDS <10, and an IADL score of < 8

The material below describes the neuropsych instruments in detail and should be moved above the definitions of HAND categories that are dervied from them.

I counted 6 domains in the MOCA, not 5 as stated

MoCA assesses six key neurocognitive domains: 1) visuospatial-executive with a total of five points comprising of clock drawing, trail making B task, and three-dimension cube copy; 2) naming of unfamiliar animals for a maximum of three points for accurate naming of each animal, language with the maximum of three points attained from sentence repetition for two points and a phonemic fluency task for one point; 3) short-term memory assessing delayed recall of words for a maximum of 5 points; 4) abstraction (verbal abstraction) for a maximum of 2 points; 5) attention and calculation assessing digits forward and backward, target detection using tapping and serial 7s subtraction for a maximum of 6 points; and 6) orientation of time and space for a maximum of 6 points(21).

its good pooled sensitivity of 0.90 [95% confidence interval (CI), 0.88-0.91] and overall specificity of 0.96 (95% CI, 0.95-0.97) under summary receiver operation(23). Compared to MoCA, which is more sensitive in screening milder forms of HAND, IHDS is more sensitive in screening for severe form of HAND(24).

The Lawton Instrumental activity of daily living (IADL) assessed the functional status of the patient's capacity for self-care, which is usually impaired among patients diagnosed with HIV-associated dementia. The scale assesses one's ability for daily tasks such as laundry, handling finances, and using a telephone. Measuring eight domains of functioning provides early warning signs of functional decline and can be administered in 10 to 15 minutes. The good performance of IADLs is used as an indicator of corresponding health cognitive status with reliable integrity (10–12)

Independent variables:

Major depressive disorders and substance use and related disorders: The standardized instrument MINI International Neuropsychiatry Interview Schedule (MINI) was used to assess these variables. MINI has acceptably high validation and reliability scores. It can also be administered much shorter (mean 18.7 + 11.6 min, median 15 min) after a brief training session, clinicians can use it, and lay interviewers require more extensive training(26). We only used the sub-scale of MDD and substance use disorders in the M.I.N.I because of their direct influence on neurocognitive performance in the HIV population(27–30).

Socio-demographic and clinical profiles were also included as explanatory variables; these include age (in years), marital status, gender, occupation (formal employment or no formal employment), years of formal education, living arrangement, most recent CD4+ count (cells/mm3), current viral load (detectable at ≥ 40 copies/ml), Hepatitis C virus (HCV) infection screening and Hepatitis B virus (HBV), type of ART regimen, HIV/AIDS clinical staging as per WHO criteria, duration of ART use (in years), Hemoglobin (HB) concentration in (mmol/L) and body mass index (BMI measured in Kg/m^2.

Data collection and analysis:

The evidence-based researcher-designed questionnaire collected sociodemographic and clinical information of interest, while psychiatric diagnoses were assessed using MINI. Two bilingual groups were consulted to translate MoCA to Swahili and then back-translated to English while maintaining a similar meaning. Interviews were conducted by trained research assistants who were medical doctors and took over forty-five working days to complete the assessment of 397 participants.

Data were analyzed using SAS version 9.4. Descriptive statistics such as frequency and percentage to describe the categorical variables, while mean and standard deviation (SD) or median and interquartile ranges (IQR) were used for continuous variables and presented using tables and figures where appropriate. A Chi-square test was computed to determine the association between socio-demographic, clinical variables, and neurocognitive decline. At the same time,

a t-test was used to determine the mean difference in neurocognitive scores between participants with HAND versus those without HAND across all cognitive domains for both MoCA and IHDS (See table 3). Unadjusted binary logistic regression was done for preliminary analysis of factors associated with HAND and adjusted for variables that reached an overall significance level of < 20% (p-value < 0.2). As most participants were negative for HBV/HCV and used only one type of ART regimen {Tenofovir (TDF) +Lamivudine (3TC) +Dolutegravir (DTG)}, these variables were not included in the logistic regression.

Ethical considerations and concerns

The study was approved by the local IRB of the Dodoma University Ethical and Research Committee with reference UDOM/ DRP/134/VOL V/91 once the proposal was fully established and merited approval. Trained research assistants who are medical doctors at the level of registrar provided participants with accurate, detailed information regarding the study. If a participant suffered from a neurological, psychiatric, or any other medical disorder needing urgent treatments, they were referred to specialized care based on the locally agreed protocols.

Written informed consent forms were provided for those who could read and write; otherwise witnessed verbal informed consent was used as an alternative. No minor was included in the study, if a participant could not consent due to medical or any other reason, a custodian or close relative provided consent.

Results

Socio-demographic and clinical characteristics of the study population by HAND

Out of 397 participants, the majority (69.5%) were women and significantly more men were neurocognitively impaired compared to women ($p<0.0001$). The mean age of the study population was 41.9 (12.6) years compared to 43.8 (11.8) and 38.8 (13.4) years for those with and without HAND respectively (p, 0.001).

Other factors significantly associated with HAND included occupation, marital status, and education level ($p<0.001$). As for the clinical profile, only non-adherence to ART had significantly higher proportions of HAND compared to those with good adherence ($p=0.050$).

Table 2: Baseline clinical profile of the study participants, N (397).

Prevalence of HIV-associated neurocognitive disorders by severity

Out of three hundred and ninety-seven participants, 58.95% met the criteria for HAND on both MoCA and IHDS, of which 58.19% had either asymptomatic neurocognitive impairment (ANI) or mild neurocognitive impairment (MNI). In

contrast, 0.76% had a severe form known as HIV-associated dementia (HAD), see figure 1. Furthermore, there were 315(79.35%) and 266(67%) participants who met exclusive criteria of neurocognitive decline for HIDS and MoCA, respectively.

Figure 1: prevalence of HAND

Table 3: pattern of neurocognitive performance based on HAND and IHDS

The mean neurocognitive performance based on MoCA was 19.7 for the study population, while the participants with HAND had significantly lower scores of 16.7 compared to those without HAND, 24.0, $p<0.001$. The mean

neurocognitive performance based on IHDS was 7.9 for the study population, while the participants with HAND had significantly lower scores of 6.9 compared to those without HAND, 9.3, p<0.001. Furthermore, participants with HAND have significantly poorer performance in each cognitive domain on both MoCA and IHDS.

Table 4: Factors associated with HIV-associated neurocognitive disorders

Logistic regression found that being female (AOR: 2.7 95%CI: 4.5, 1.59) p=0.002) was associated with poor cognitive functioning. Higher age category was significantly associated with neurocognitive impairment under unadjusted analysis although the association remained significant only for those with 55+ years and above (AOR: 3.5 95% CI: 1.1, 11.6, p=0.041) under adjusted analysis. Adherence to ART (AOR: 0.40(95%CI: 0.164 0.98), p=0.044) having attained primary education (AOR: 0.34.0 %CI: 0.14, 0.77 p=0.010) or secondary education (AOR: 0.091(95%CI: 0.035, 0.24) p<0.001) compared to having no formal education were all associated with favorable cognitive performance.

Discussion

The high prevalence (58.2 %) of either asymptomatic or mild neurocognitive impairment that we found in our HIV+ population was associated with advanced age, female sex, less education and ART adherence. Only about 1% met the criteria for HIV-associated dementia. This pattern reflects the effectiveness of HAART in reducing the severe forms of HAND while the prevalence of milder forms remains high, ranging between 21% to 81%(31,32).

A higher prevalence of neurocognitive disorders in HIV has been reported elsewhere in sub-Saharan Africa. For example, prevalence of 81.1% was reported from Eldoret, Kenya(24), 68.4% in Dar es Salaam Tanzania(33), and 68.6% in Kampala Uganda(34). The use of a single screening instrument for neurocognitive function in most of these studies compared to the requirement to meet the criteria on both MoCA and IHDS in our study could explain the difference in prevalence rates. Conversely, a lower prevalence of HAND is reported when a comprehensive neuropsychological battery based on Frascati criteria is used; a meta-analysis shows a global prevalence of HAND to be 44.9%, with the specific a distribution of 26.2%, 8.5%, and 2.1% for ANI, MND and HAD, respectively(35). Although 41% of the population did not meet the criteria for HAND, the overall mean neurocognitive scores of the whole population were below the cutoff

point for IHDS (24.00) but not on MoCA (9.31). Furthermore, the participants with HAND had significantly lower scores than those without HAND across all cognitive domains on both tools, suggesting diffuse brain involvement related to global cognitive impairment. In HIV infection, subcortical brain structures are particularly vulnerable, especially at the early stages(36); however, the cortical areas are also not spared as the infection progresses and may be linked with motor decline and subsequent cognitive dysfunction(37).

The mean scores of the study population were below the set cut-off point on both IHDS and MoCA; also, more participants (79%) met the criteria for neurocognitive decline on IHDS compared to 67% on MoCA. This observation may imply the distinct capacity of IHDS to assess specific cognitive domains such as the motor and psychomotor speed that were poorly performed by our participants in general but are not assessed in the MoCA. These specific deficits may suggest an involvement in subcortical structures which are particularly vulnerable in HIV infection and may indicate severe cognitive impairment. Higher proportion of positive screening on IHDS than MoCA may also suggests that the cognitive scores were skewed towards a more severe cognitive impairment within the spectrum of ANI to MND regardless of just about 1% meeting the criteria for most severe form of HAND known as HIV associated Dementia(24).

Regression analysis found that being female and older were significantly associated with neurocognitive impairment. Women had

poor cognitive functioning compared to men, especially in speed of information processing (SIP), memory, and motor functions(38).

he sex difference in neurocognitive impairment is attributed to mental health, and other comorbidities and biological factors(38). Cognitive reserve is a factor in determining neurocognitive impairment when the brain is traumatized (39,40). Compared to men, women living with HIV tend to have lower cognitive reserve. (41,42).

Factors that may account for this difference include low education, poverty, depression, barriers to healthcare and early life trauma which are more common among women than men(43,44).

Stress and early life trauma as well as mental health disorders tend to affect neurocognitive functioning more in women living with HIV than in men; evidence supports that anxiety, depression, PTSD, and perceived stress are all associated with deficits in memory, learning, and attention(45–48).

The gender differences in neurocognitive performance in HIV could also be linked to endocrinological and immunological factors. Generally, sex steroids, including estradiol, progesterone, and testosterone can influence neurocognitive performance in a healthy population and their optimal levels are linked to good verbal performance in females and visual-spatial performance in males(38). Specific female-related factors, including menstrual

cycle, menopause, and pregnancy, bring complex dynamics that influence neurocognitive performance(49) even more. In HIV, through viral suppression and replications, estradiol is directly linked to transcriptional activities that affect neurocognitive activities(50,51). Indeed, both progesterone and estradiol impact immunomodulatory mechanisms through chemokine and cytokines, which also influence neurocognitive functioning(52). Emerging evidence highlights sex differences in immune function and sex-specific genetic determinants of immune response to HIV infection (53) with variation in pathogenesis-related to chronic immune activation linked to HIV-induced neurotoxicity and eventual neurocognitive decline(54,55). Although evidence is not well established, the sex difference in monocyte-associated inflammatory biomarkers has been observed. For example, females have higher levels of sCD163 than males (43–45). Also, Neopterine, a marker of cellular immune activation whose CSF levels are generally associated with NCI, their plasma concentration appears to be associated with NCI only in WLWH but not in MLWH(58–61).

In this study, age was also significantly associated with poor neurocognitive decline. While the overall age group was associated with poor cognitive performance at univariate analysis, only in those above 55 years old was age significantly associated with a neurocognitive impairment when adjusted for confounding variables. As HIV/AIDS is now considered a chronic disease, the interaction between HIV and aging affects the brain, and neurocognitive

functions become more apparent(62). Our findings support the previous observation that advanced age is among the factors associated with reduced neurocognitive decline and vulnerability to HAND in the HIV population(63–66). The pathogenesis of neurocognitive decline could be partly incluenced by the additive impact of aging and HIV (67,68). Emerging evidence also supports the synergistic effect of the two risk factors: eg, acceleration memory deficits ove rone year are found among older but not a younger people with HIV population (69,70)

The two factors that were protective against neurocognitive impairment are higher educational achievement and adherence to ART. Similar to previous studies, a higher level of education was associated with better cognitive performance. Although there were fewer participants with an advanced level of education, the higher achievement was associated with the better the cognitive performance. Advanced education is thought to improve cognitive reserve and delay neurocognitive decline and functional expression of HIV-related neurodegenerative processes (71–73). Furthermore, the effect of education is reflected when using cognitive screening tools, including MoCA and MMSE, which are sensitive to educational and socioeconomic factors; for this reason, lower cut-off points or the addition of a point is recommended for those patients with less than twelve years of formal education.

The relationship between neurocognitive functioning and

adherence to ART is increasingly becoming an area of interest.

Given that the ART adherence rate in sub-Saharan Africa is below the recommended 95%, the reciprocal relation between neurocognitive impairment and ART adherence becomes a significant issue of concern. Our study showed that suboptimal adherence was significantly associated with neurocognitive impairment. Other studies have also shown that neurocognitive decline is a major predictor of poor adherence to ART(74). However, delineating whether or not poor adherence caused poor cognitive function and/or vice versa is unclear.

PLWH with major depresison (MDD) had 35% more odds of neurocognitive decline than those without. The
main explanation for this observation is the low prevalence of MDD, which had to be computed against neurocognitive decline and controlled for confounding variables. **(Not clear how these two observations are connected?)**
and IHDS criteria had to be met and thus further lowering the prevalence of Furthermore, to meet the criteria of HAND, both MoCA
HAND to compute against the low prevalence of MDD. The negative impact of MDD on cognitive performance is supported by the previous analysis of the same data which was screened by MoCA alone and showed a strong negative association between the two(75).

The study had several limitations; first, cross-sectional studies are unable to delineate the temporal relationship between HAND and the

explanatory variables. Secondly, using MoCA and IHDS instead of the gold standard comprehensive neuropsychological assessment lowers the diagnostic accuracy and ability to fully categorize HAND into ANI, MND, and HAD. However, both MoCA and IHDS are recommended instruments in settings where the use of ideal gold standard assessment is not feasible. Furthermore, to improve the sensitivity and specificity, the HAND diagnosis was only met if both MoCA and IHDS scores were below the set cutoff points. Also, using IADL improved the ability to identify HAD, albeit the inability to distinguish ANI from MND remained a limitation.

IRB & Ethics Approvals:

The study was approved by the Dodoma University Ethical and Research Committee once the proposal was fully established and merited approval.

Accurate information about the study was provided to the participants by the trained research assistants who were medical doctors at the registrar level. In case a participant had a psychiatric, neurological, or any other medical disorder requiring treatment, they were referred to CTC clinicians, neurologists, or mental health clinicians according to a locally agreed protocol. Where participants could not consent due to cognitive impairment or any other reason, assent was sought from a custodian who had to be a close relative.

Acknowledgments

The author acknowledges the Fogarty International Center of the National Institutes of Health for funding the study under award number D43TW009775. The

content is solely the author's responsibility and does not necessarily reflect the official views of the National Institute of Health.

Reference

1. Shapshak P, Kangueane P, Fujimura RK, Commins D, Chiappelli F, Singer E, et al. Editorial neuroAIDS review. AIDS Lond Engl. 2011 Jan 14;25(2):123–41.

2. Price RW. Neurological complications of HIV infection. Lancet Lond Engl. 1996 Aug 17;348(9025):445–52.

3. Antinori A, Arendt G, Becker JT, Brew BJ, Byrd DA, Cherner M, et al. Updated research nosology for HIV-associated neurocognitive disorders. Neurology. 2007 Oct 30;69(18):1789–99.

4. Gisslén M, Price RW, Nilsson S. The definition of HIV-associated neurocognitive disorders: are we overestimating the real prevalence? BMC Infect Dis. 2011 Dec 28;11:356.

5. Wang Y, Liu M, Lu Q, Farrell M, Lappin JM, Shi J, et al. Global prevalence and burden of HIV-associated neurocognitive disorder: A meta-analysis. Neurology. 2020 Nov 10;95(19):e2610–21.

6. Masliah E, DeTeresa RM, Mallory ME, Hansen LA. Changes in pathological findings at autopsy in AIDS cases for the last 15 years. AIDS. 2000 Jan 7;14(1):69–74.

7. Gannon P, Khan MZ, Kolson DL. Current understanding of HIV-associated neurocognitive disorders pathogenesis. Curr Opin Neurol. 2011 Jun;24(3):275–83.

8. Kramer-Hämmerle S, Rothenaigner I, Wolff H, Bell JE, Brack-Werner R. Cells of the central nervous system as targets and reservoirs of the human immunodeficiency virus. Virus Res. 2005 Aug;111(2):194–213.

9. Saylor D, Dickens AM, Sacktor N, Haughey N, Slusher B, Pletnikov M, et al. HIV-associated neurocognitive disorder — pathogenesis and prospects for treatment. Nat Rev Neurol. 2016 Apr;12(4):234–48.

10. Gelman BB. Neuropathology of HAND With Suppressive Antiretroviral Therapy: Encephalitis and Neurodegeneration Reconsidered. Curr HIV/AIDS Rep. 2015 Jun;12(2):272–9.

11. Cysique LA, Brew BJ. Vascular cognitive impairment and HIV-associated neurocognitive disorder: a new paradigm. J Neurovirol. 2019 Oct 1;25(5):710–21.

12. Sacktor N, Skolasky RL, Seaberg E, Munro C, Becker JT, Martin E, et al. Prevalence of HIV-associated neurocognitive disorders in the Multicenter AIDS Cohort Study. Neurology. 2016 Jan 26;86(4):334–40.

13. Bloch M, Kamminga J, Jayewardene A, Bailey M, Carberry A, Vincent T, et al. A Screening Strategy for HIV-Associated Neurocognitive Disorders That Accurately Identifies Patients Requiring Neurological Review. Clin Infect Dis Off Publ Infect Dis Soc Am. 2016 Sep 1;63(5):687–93.

14. Lj H, R L, M D, J M, Fc L, R G, et al. Multicenter European Prevalence Study of Neurocognitive Impairment and Associated Factors in HIV Positive Patients. AIDS

Behav [Internet]. 2018 May [cited 2022 Jun 28];22(5). Available from: https://pubmed.ncbi.nlm.nih.gov/28144792/

15. Ellis RJ, Badiee J, Vaida F, Letendre S, Heaton RK, Clifford D, et al. CD4 nadir is a predictor of HIV neurocognitive impairment in the era of combination antiretroviral therapy. AIDS Lond Engl. 2011 Sep 10;25(14):1747–51.

16. Census General Report-2012PHC.pdf [Internet]. [cited 2022 Apr 7]. Available from: http://tanzania.countrystat.org/fileadmin/user_upload/countrystat_fenix/congo/docs/Census%20General%20Report-2012PHC.pdf

17. Carson N, Leach L, Murphy KJ. A re-examination of Montreal Cognitive Assessment (MoCA) cutoff scores. Int J Geriatr Psychiatry. 2018;33(2):379–88.

18. Masika GM, Yu DSF, Li PWC. Accuracy of the Montreal Cognitive Assessment in Detecting Mild Cognitive Impairment and Dementia in the Rural African Population. Arch Clin Neuropsychol Off J Natl Acad Neuropsychol. 2021 Apr 21;36(3):371–80.

19. Koenig N, Fujiwara E, Gill MJ, Power C. Montreal Cognitive Assessment Performance in HIV/AIDS: Impact of Systemic Factors. Can J Neurol Sci J Can Sci Neurol. 2015 Dec 4;1–6.

20. Rosca EC, Albarqouni L, Simu M. Montreal Cognitive Assessment (MoCA) for HIV-Associated Neurocognitive Disorders. Neuropsychol Rev. 2019 Sep 1;29(3):313–27.

21. Nasreddine ZS, Phillips NA, Bédirian V, Charbonneau S, Whitehead V, Collin I, et al. The Montreal Cognitive Assessment, MoCA: a brief screening tool for mild cognitive impairment. J Am Geriatr Soc. 2005 Apr;53(4):695–9.

22. Utility of the HIV Dementia Scale (HDS) in identifying HIV dementia in a South African sample. J Neurol Sci. 2008 Jun 15;269(1–2):62–4.

23. Hu X, Zhou Y, Long J, Feng Q, Wang R, Su L, et al. Diagnostic accuracy of the International HIV Dementia Scale and HIV Dementia Scale: A meta-analysis. Exp Ther Med. 2012 Oct;4(4):665–8.

24. Mohamed AA, Oduor C, Kinyanjui D. HIV-associated neurocognitive disorders at Moi teaching and referral hospital, Eldoret, Kenya. BMC Neurol. 2020 Jul 14;20(1):280.

25. Gold DA. An examination of instrumental activities of daily living assessment in older adults and mild cognitive impairment. J Clin Exp Neuropsychol. 2012;34(1):11–34.

26. Amorim P. Mini International Neuropsychiatric Interview (MINI): validação de entrevista breve para diagnóstico de transtornos mentais. Braz J Psychiatry. 2000 Sep;22(3):106–15.

27. Moore DJ, Blackstone K, Woods SP, Ellis RJ, Atkinson JH, Heaton RK, et al. Methamphetamine use and neuropsychiatric factors are associated with antiretroviral non-adherence. AIDS Care. 2012 Dec 1;24(12):1504–13.

28. Hendershot CS, Stoner SA, Pantalone DW, Simoni JM. Alcohol Use and Antiretroviral Adherence: Review and Meta-Analysis. JAIDS J Acquir Immune Defic Syndr. 2009 Oct;52(2):180–202.

29. Thaler NS, Sayegh P, Kim MS, Castellon SA, Hinkin CH. Interactive Effects of Neurocognitive Impairment and Substance Use on Antiretroviral Non-adherence in HIV Disease. Arch Clin Neuropsychol. 2015 Mar 1;30(2):114–21.

30. Hinkin CH, Castellon SA, Durvasula RS, Hardy DJ, Lam MN, Mason KI, et al. Medication adherence among HIV+ adults. Neurology. 2002 Dec 24;59(12):1944–50.

31. McDonnell J, Haddow L, Daskalopoulou M, Lampe F, Speakman A, Gilson R, et al. Minimal cognitive impairment in UK HIV-positive men who have sex with men: effect of case definitions and comparison with the general population and HIV-negative men. J Acquir Immune Defic Syndr 1999. 2014 Oct 1;67(2):120–7.

32. Rosenthal LS, Skolasky RL, Moxley RT, Roosa HV, Selnes OA, Eschman A, et al. A novel computerized functional assessment for human immunodeficiency virus-associated neurocognitive disorder. J Neurovirol. 2013 Oct;19(5):432–41.

33. Nyundo AA, Ndetei DM, Othieno CJ, Mathai AM. Neurocognitive correlates of the use of combined Antiretroviral Therapy among HIV-infected adults attending care and treatment center at Muhimbili National Hospital, Dar es Salaam, Tanzania: An analytical crosssectional study. Tanzan Med J [Internet]. 2015 [cited 2022 May 28];27(1). Available from: https://www.ajol.info/index.php/tmj/article/view/129268

34. Nakasujja N, L Skolasky R, Musisi S, Allebeck P, Robertson K, Ronald A, et al. Depression symptoms and cognitive function among individuals with advanced HIV infection initiating HAART in Uganda. BMC Psychiatry. 2010 Jun 10;10(1):44.

35. Wei J, Hou J, Su B, Jiang T, Guo C, Wang W, et al. The Prevalence of Frascati-Criteria-Based HIV-Associated Neurocognitive Disorder (HAND) in HIV-Infected Adults: A Systematic Review and Meta-Analysis. Front Neurol [Internet]. 2020 [cited 2022 May 28];11. Available from: https://www.frontiersin.org/article/10.3389/fneur.2020.581346

36. Küper M, Rabe K, Esser S, Gizewski ER, Husstedt IW, Maschke M, et al. Structural gray and white matter changes in patients with HIV. J Neurol. 2011 Jun 1;258(6):1066–75.

37. Zhou Y, Li R, Wang X, Miao H, Wei Y, Ali R, et al. Motor-related brain abnormalities in HIV-infected patients: a multimodal MRI study. Neuroradiology. 2017 Nov 1;59(11):1133–42.

38. Rubin LH, Neigh GN, Sundermann EE, Xu Y, Scully EP, Maki PM. Sex Differences in Neurocognitive Function in Adults with HIV: Patterns, Predictors, and Mechanisms. Curr Psychiatry Rep. 2019 Sep 14;21(10):94.

39. Y S. What is cognitive reserve? Theory and research application of the reserve concept. J Int Neuropsychol Soc JINS [Internet]. 2002 Mar [cited 2022 Jun 7];8(3). Available from: https://pubmed.ncbi.nlm.nih.gov/11939702/?dopt=Abstract

40. Stern Y, Gurland B, Tatemichi TK, Tang MX, Wilder D, Mayeux R. Influence of education and occupation on the incidence of Alzheimer's disease. JAMA. 1994 Apr 6;271(13):1004–10.

41. Tsai AC, Burns BFO. Syndemics of psychosocial problems and HIV risk: A systematic review of empirical tests of the disease interaction concept. Soc Sci Med 1982. 2015 Aug;139:26–35.

42. Singer M. AIDS and the health crisis of the U.S. urban poor; the perspective of critical medical anthropology. Soc Sci Med 1982. 1994 Oct;39(7):931–48.

43. Basso MR, Bornstein RA. Estimated premorbid intelligence mediates neurobehavioral change in individuals infected with HIV across 12 months. J Clin Exp Neuropsychol. 2000 Apr;22(2):208–18.

44. R F, En M, P S, Oa S, Ba C, Jt B, et al. Psychosocial risk factors of HIV morbidity and mortality: findings from the Multicenter AIDS Cohort Study (MACS). J Clin Exp Neuropsychol [Internet]. 2003 Aug [cited 2022 Jun 7];25(5). Available from: https://pubmed.ncbi.nlm.nih.gov/12815503/?dopt=Abstract

45. Maki PM, Rubin LH, Valcour V, Martin E, Crystal H, Young M, et al. Cognitive function in women with HIV: findings from the Women's Interagency HIV Study. Neurology. 2015 Jan 20;84(3):231–40.

46. Rubin LH, Pyra M, Cook JA, Weber KM, Cohen MH, Martin E, et al. Post-traumatic stress is associated with verbal learning, memory, and psychomotor speed in HIV-infected and HIV-uninfected women. J Neurovirol. 2016 Apr 1;22(2):159–69.

47. Rubin LH, Cook JA, Weber KM, Cohen MH, Martin E, Valcour V, et al. The association of perceived stress and verbal memory is greater in HIV-infected versus HIV-uninfected women. J Neurovirol. 2015 Aug 1;21(4):422–32.

48. Rubin LH, Sundermann EE, Cook JA, Martin EM, Golub ET, Weber KM, et al. Investigation of menopausal stage and symptoms on cognition in human immunodeficiency virus-infected women. Menopause N Y N. 2014 Sep;21(9):997–1006.

49. Sherwin BB. Estrogen and cognitive functioning in women: lessons we have learned. Behav Neurosci. 2012 Feb;126(1):123–7.

50. Das B, Dobrowolski C, Luttge B, Valadkhan S, Chomont N, Johnston R, et al. Estrogen receptor-1 is a key regulator of HIV-1 latency that imparts gender-specific restrictions on the latent reservoir. Proc Natl Acad Sci U S A. 2018 Aug 14;115(33):E7795–804.

51. Szotek EL, Narasipura SD, Al-Harthi L. 17β-Estradiol inhibits HIV-1 by inducing a complex formation between β-catenin and estrogen receptor a on the HIV promoter to suppress HIV transcription. Virology. 2013 Sep 1;443(2):375–83.

52. Devadas K, Biswas S, Ragupathy V, Lee S, Dayton A, Hewlett I. Modulation of HIV replication in monocyte derived macrophages (MDM) by steroid hormones. PloS One. 2018;13(1):e0191916.

53. Schmiedel BJ, Singh D, Madrigal A, Valdovino-Gonzalez AG, White BM, Zapardiel-Gonzalo J, et al. Impact of Genetic Polymorphisms on Human Immune Cell Gene Expression. Cell. 2018 Nov 29;175(6):1701-1715.e16.

54. Raghavan A, Rimmelin DE, Fitch KV, Zanni MV. Sex Differences in Select Non-communicable HIV-Associated Comorbidities: Exploring the Role of Systemic Immune Activation/Inflammation. Curr HIV/AIDS Rep. 2017 Dec;14(6):220–8.

55. Lipton SA. Requirement for macrophages in neuronal injury induced by HIV envelope protein gp120. Neuroreport. 1992 Oct;3(10):913–5.

56. Ticona E, Bull ME, Soria J, Tapia K, Legard J, Styrchak SM, et al. Biomarkers of inflammation in HIV-infected Peruvian men and women before and during suppressive antiretroviral therapy. AIDS Lond Engl. 2015 Aug 24;29(13):1617–22.

57. Martin GE, Gouillou M, Hearps AC, Angelovich TA, Cheng AC, Lynch F, et al. Age-associated changes in monocyte and innate immune activation markers occur more rapidly in HIV infected women. PloS One. 2013;8(1):e55279.

58. Burdo TH, Weiffenbach A, Woods SP, Letendre S, Ellis RJ, Williams KC. Elevated sCD163 in plasma but not cerebrospinal fluid is a marker of neurocognitive impairment in HIV infection. AIDS Lond Engl. 2013 Jun 1;27(9):1387–95.

59. Krebs SJ, Slike BM, Sithinamsuwan P, Allen IE, Chalermchai T, Tipsuk S, et al. Sex differences in soluble markers vary before and after the initiation of antiretroviral therapy in chronically HIV-infected individuals. AIDS Lond Engl. 2016 Jun 19;30(10):1533–42.

60. Hagberg L, Cinque P, Gisslen M, Brew BJ, Spudich S, Bestetti A, et al. Cerebrospinal fluid neopterin: an informative biomarker of central nervous system immune activation in HIV-1 infection. AIDS Res Ther. 2010 Jun 3;7:15.

61. Dunbar N, Pemberton L, Perdices M, Brew BJ. Clinical Markers of the Presence of Dementia and Neuropsychological Impairment in HIV Infection. J Neuro-AIDS. 1996;1(4):31–48.

62. Cohen RA, Seider TR, Navia B. HIV effects on age-associated neurocognitive dysfunction: premature cognitive aging or neurodegenerative disease? Alzheimers Res Ther. 2015 Apr 6;7(1):37.

63. Wendelken LA, Valcour V. Impact of HIV and aging on neuropsychological function. J Neurovirol. 2012 Aug;18(4):256–63.

64. Valcour VG, Shikuma CM, Watters MR, Sacktor NC. Cognitive impairment in older HIV-1-seropositive individuals: prevalence and potential mechanisms. AIDS Lond Engl. 2004 Jan 1;18 Suppl 1:S79-86.

65. Becker JT, Lopez OL, Dew MA, Aizenstein HJ. Prevalence of cognitive disorders differs as a function of age in HIV virus infection. AIDS Lond Engl. 2004 Jan 1;18 Suppl 1:S11-18.

66. Wilkie FL, Goodkin K, Khamis I, van Zuilen MH, Lee D, Lecusay R, et al. Cognitive functioning in younger and older HIV-1-infected adults. J Acquir Immune Defic Syndr 1999. 2003 Jun 1;33 Suppl 2:S93–105.

67. Becker JT, Maruca V, Kingsley LA, Sanders JM, Alger JR, Barker PB, et al. Factors affecting brain structure in men with HIV disease in the post-HAART era. Neuroradiology. 2012 Feb;54(2):113–21.

68. Ances BM, Ortega M, Vaida F, Heaps J, Paul R. Independent effects of HIV, aging, and HAART on brain volumetric measures. J Acquir Immune Defic Syndr 1999. 2012 Apr 15;59(5):469–77.

69. Seider TR, Luo X, Gongvatana A, Devlin KN, de la Monte SM, Chasman JD, et al. Verbal memory declines more rapidly with age in HIV infected versus uninfected adults. J Clin Exp Neuropsychol. 2014;36(4):356–67.

70. Morgan EE, Woods SP, Smith C, Weber E, Scott JC, Grant I, et al. Lower cognitive reserve among individuals with syndromic HIV-associated neurocognitive disorders (HAND). AIDS Behav. 2012 Nov;16(8):2279–85.

71. Kabuba N, Menon JA, Franklin DR, Lydersen S, Heaton RK, Hestad KA. Effect of Age and Level of Education on Neurocognitive Impairment in HIV Positive Zambian Adults. Neuropsychology. 2018 Jul;32(5):519–28.

72. Le Carret N, Lafont S, Mayo W, Fabrigoule C. The effect of education on cognitive performances and its implication for the constitution of the cognitive reserve. Dev Neuropsychol. 2003;23(3):317–37.

73. Ostrosky-Solis F, Ardila A, Rosselli M, Lopez-Arango G, Uriel-Mendoza V. Neuropsychological test performance in illiterate subjects. Arch Clin Neuropsychol Off J Natl Acad Neuropsychol. 1998 Oct;13(7):645–60.

74. Nyundo AA. Neurocognitive decline as a major predictor of nonadherence to antiretroviral therapy among adults living with HIV in Dodoma region, central Tanzania. Health Sci Rep. 2022;5(4):e669.

75. Nyundo AA, Ismail A. The influence of major depressive disorders on neurocognitive function among adults living with HIV/AIDS in a regional referral hospital in Dodoma, Tanzania. Trop Med Int Health. 2022;27(1):58–67.

Figure 1

Click here to access/download;Figure;figure 1.tif

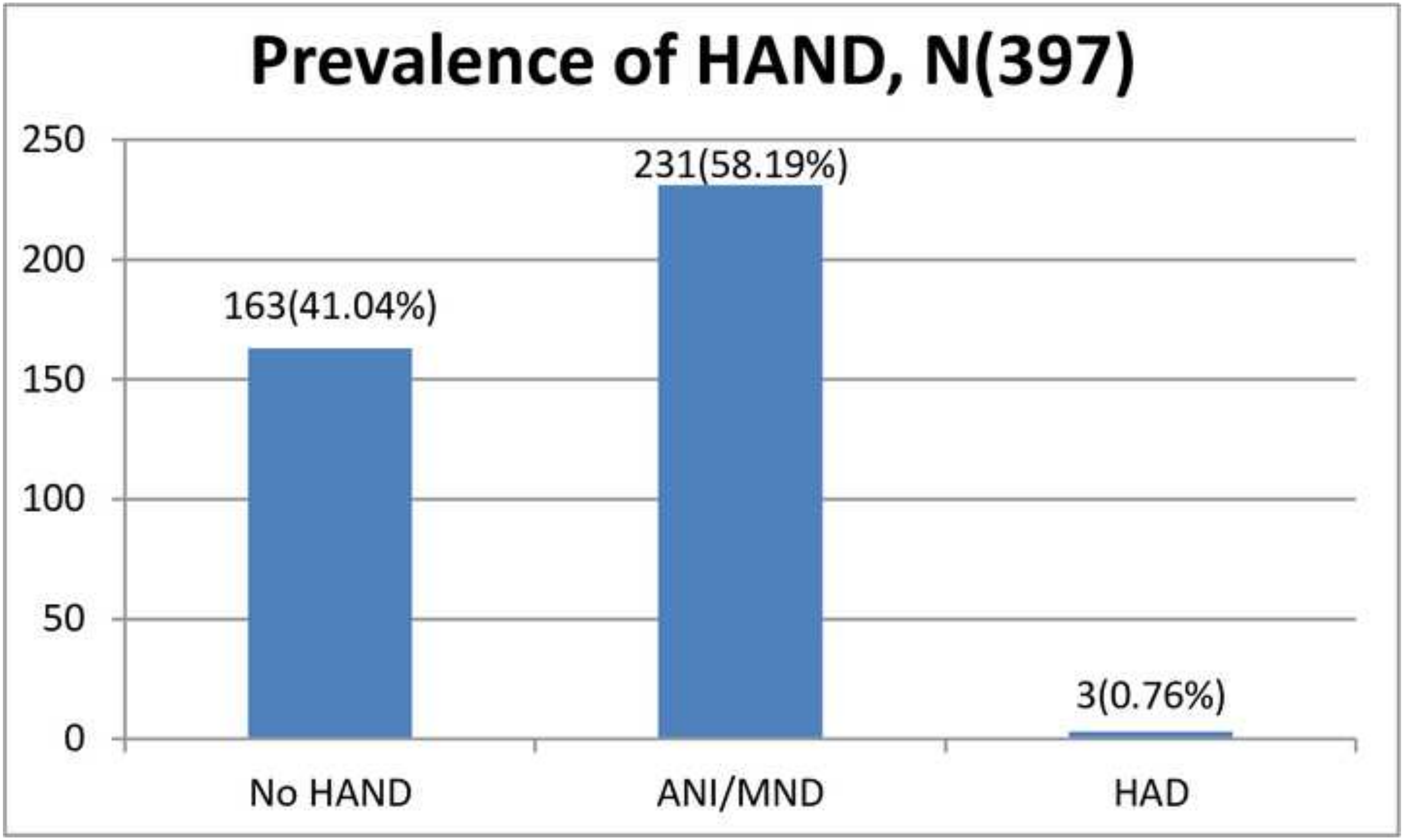

Table 1: Socio-demographic characteristics by HAND status of the study population (**N=397**)				
**Variable**	**All N (%)**	**HAND N (%)**	**No HAND N (%)**	**P-value**
**Age of the respondent**				<0.0001
Mean(SD)	41.93(12.62)	43.84(11.81)	38.81(13.35)	
≤24	43(10.83)	11 (25.58)	32(74.42)	
25-34	65(16.37)	35(53.85)	30(46.15)	
35-44	104(26.20)	64(61.54)	40(38.46)	
45-54	117(29.47)	77(65.81)	40(34.19)	
≥55	68(17.13)	47(69.12)	21(30.88)	
**Gender**				<0.0001
Male	121(30.48)	51(42.15)	70(57.85)	
Female	276(69.52)	183(66.30)	93(33.70)	
**Marital status**				<0.0001
Married/Cohabiting	162(40.81)	98(60.49)	64(39.51)	
Never married	80(20.15)	26(32.50)	54(67.50)	
Divorced/Separated	89(22.42)	68(76.40)	21(23.60)	
Widowed	66(16.62)	42(63.64)	24(36.36)	
**Years of formal education**				
No formal education	55(13.85)	47(85.45)	8(14.55)	<0.0001
Primary complete/incomplete	245(61.71)	161(65.71)	84(84.29)	
Secondary education ≥ 8-years	97(24.43)	26(26.80)	71(73.20)	
**Occupation status**				
Self-employed/unemployed	350(88.16)	220(62.86)	130(37.14)	<0.0001
Employed	47(11.84)	14(29.79)	33(70.21)	
**Living arrangement**				
Lives alone	88(22.17)	50(56.82)	38(43.18)	0.8056
Lives with nursing staff/caregiver	71(17.88)	43(60.56)	28(39.44)	
Lives with spouse	150(37.78)	92(61.33)	58(38.67)	
Lives with friends/other	88(22.17)	49(55.68)	39(44.32)	

Table 2: Baseline clinical profile and major depressive disorders of the study participants, N(397)				
**Variable**	**All N (%)**	**HAND N (%)**	**No HAND N (%)**	**Chi-square/Student t-test (p-value)**
**Major depressive disorders**				0.1763
Yes	22(5.54)	16(72.73)	6(27.27)	
No	375(94.46)	218(58.13)	157(41.87)	
**Substance use/disorder**				0.8661
Yes	45(11.34)	26(57.78)	19(42.22)	
No	352(88.66)	208(59.09)	144(40.91)	
**WHO clinical staging**				0.379
I	84(21.16)	43 (51.19)	41 (48.81)	
Ii	154(38.79)	96 (62.34)	58 (37.66)	
Iii	115(28.97)	70 (60.87)	45 (39.13)	
IV	44(11.08)	25 (56.82)	19 (43.18)	
**ART regime**				0.6526
TDF + 3TC + DTG	382(96.22)	226 (59.16)	156 (40.84)	
Others	15(3.78)	8 (53.33)	7 (46.67)	
**Most recent CD4 count**				0.3075
<200	53(13.35)	28 (52.83)	25 (47.17)	
200-499	148(37.28)	94 (63.51)	54 (36.49)	
≥500	196(49.37)	112 (57.14)	84 (42.86)	
**Current viral load**				0.7812
Undetected	88(22.17)	53 (60.23)	35 (39.77)	
Detected	309(77.83)	181 (58.58)	128 (41.42)	
**Adherence to ART**				0.0505
Non-adherence	41(10.33)	30(73.17)	11(26.83)	
Adherence	356(89.67)	204(57.30)	152(42.70)	
**Duration of ART use: Mean(SD)**	6.12(4.60)	6.08(4.42)	6.18(4.85)	0.8369
**Haemoglobin(Hb) concentration**	12.40(2.57)	12.42(2.52)	12.38(2.66)	0.8971

Click here to access/download;Figure;Table 3.tif

Table 3: Pattern of neurocognitive performance by cognitive domain based on MoCA and IHDS, N(397).				
**Domain**	All N(397)	HAND (N=234)	No HAND(N=163)	P-value
**MoCA**	19.67(5.15)	16.65 (3.98)	24.00 (3.15)	<0.0001
Visual executive	2.66(1.70)	1.80(0.88)	3.90 (1.31)	<0.0001
Naming	2.29 (0.85)	2.00 (0.88)	2.71 (0.57)	<0.0001
Attention	3.29 (1.74)	2.45 (1.44)	4.50 (1.39)	<0.0001
Language	0.79 (0.88)	0.47 (0.69)	1.24 (0.93)	<0.0001
Abstraction	1.81 (0.49)	1.72 (0,58)	1.95 (0.27)	<0.0001
Delayed recall	3.01(1.48)	2.49 (1.44)	3.75 (1.20)	<0.0001
Orientation	5.82 (0.52)	5.73 (0.64)	5.95 (0.22)	<0.0001
**IHDS**	7.88 (2.09)	6.89 (1.69)	9.31 (1.78)	<0.0001
Motor speed	2.14 (0.84)	1.87(0.71)	2.53 (0.85)	<0.0001
Psychomotor speed	2.66 (1.09)	2.28 (1.02)	3.21 (0.95)	<0.0001
Memory-recall	3.08 (0.96)	2.74 (0.74)	3.56 (0.72)	<0.0001

Lives with nursing staff/caregiver	1.167(0.618, 2.205)	0.6338		
Lives with spouse	1.206 (0.706, 2.058)	0.4933		
Lives with friends/other	0.955 (0.526, 1.732)	0.8792		
**Current MDD**		0.1833		0.6082
Yes	1.919(0.735, 5.015)		1.358(0.422,4.372)	
No	Ref			
**Substance use/disorder**		0.8656		
Yes	0.947(0.505, 1.776)			
No				
**Current WHO staging**		0.3826		
Stage I	Ref			
Stage II	1.578 (0.922, 2.702)	0.0964		
Stage III	1.483 (0.840, 2.619)	0.1742		
Stage IV	1.255 (0.602, 2.614)	0.5448		
**Most recent CD4+ count**		0.3089		
<200	Ref			
200-499	1.554(0.824, 2.932)	0.1733		
≥500	1.190(0.647, 2.189)	0.5747		
**Current viral load**		0.7820		
Undetected	Ref			
Detected	1.071 (0.660, 1.736)			
**Adherence to ART**				
Optimal adherence	0.492(0.239, 1.013)	0.0543	0.401(0.164, 0.977)	0.0443
Sub-optimal adherence	Ref			
**Duration of ART use**	1.004(0.961,1.049)	0.8515		

Lives with nursing staff/caregiver	1.167(0.618, 2.205)	0.6338		
Lives with spouse	1.206 (0.706, 2.058)	0.4933		
Lives with friends/other	0.955 (0.526, 1.732)	0.8792		
**Current MDD**		0.1833		0.6082
Yes	1.919(0.735, 5.015)		1.358(0.422,4.372)	
No	Ref			
**Substance use/disorder**		0.8656		
Yes	0.947(0.505, 1.776)			
No				
**Current WHO staging**		0.3826		
Stage I	Ref			
Stage II	1.578 (0.922, 2.702)	0.0964		
Stage III	1.483 (0.840, 2.619)	0.1742		
Stage IV	1.255 (0.602, 2.614)	0.5448		
**Most recent CD4+ count**		0.3089		
<200	Ref			
200-499	1.554(0.824, 2.932)	0.1733		
≥500	1.190(0.647, 2.189)	0.5747		
**Current viral load**		0.7820		
Undetected	Ref			
Detected	1.071 (0.660, 1.736)			
**Adherence to ART**				
Optimal adherence	0.492(0.239, 1.013)	0.0543	0.401(0.164, 0.977)	0.0443
Sub-optimal adherence	Ref			
**Duration of ART use**	1.004(0.961,1.049)	0.8515		

line numbered manuscript

Click here to access/download
Supporting Information
Line numbered Manuscript HAND plos one.docx
