# Supplementary material for: Correlates of the HIV-associated neurocognitive disorders among adults living with HIV in Dodoma region, central Tanzania: A cross-sectional study
Source: PLoS One. 2023 May 25;18(5):e0285761. doi: 10.1371/journal.pone.0285761 (PMC10212098; doi:10.1371/journal.pone.0285761)
Supplement: Attachment — Submitted filename: Response to reviewers.docx [file pone.0285761.s003.docx]

**Response to reviews**

Comments from reviewer # 1

The author has accepted the edits from the PDF of the reviewer # 1 made amendments

1. The study design was not well described.

- The study design is now corrected as per reviewer’s suggestions, see highlighted line number 84.

2. They use two standardized instruments to assess cognition that have been used in other studies in Africa and use cutoffs that appear appropriate or less educated populations, but do not discuss why they did not use results of a prior study of cognition in the general rural population (ref 18) to assess their participants’ performance.

- The justification of not using the results is given in the operational description of HAND in the methods section; see line number 120-128.

3. They did not discuss the details of the educational levels, training and assessment of competence of the interviewers/testers.

- This is now discussed in detail; see highlighted line number 198-205.

4. They assess and report bivariate analysis of risk factors for HAND, but do not appear to have performed multivariate modeling of these factors which are likely to be correlated. Such as analysis is standard for this sort of study.

- Thank you for this comment. I would like to clarify that the multivariate modeling was computed; the analysis is now clarified in highlighted in line number 217-221 and also table 4 showing univariate logistic analysis (unadjusted) and multivariate/multivariable logistic regression (adjusted) analysis.

5. Several times the authors refer to cognitive "decline" (implying a change from baseline to follow-up) when they mean "impairment" (less than expected normative performance for the population) in this cross sectional study.

- The term now used throughout the document is “impairment” unless citing references that used the specific term.

6. Data in results is reported to more decimal places (4) than is justified by either the precision of the estimate or needs of the reader.

- With exception to the statistical p-values which are in three decimal places, while the rest of the numbers were reduced to single decimal place. The descriptions highlighted in line number 209-212 while changes are made throughout all the tables.

7. The paragraph in the discussion beginning with "PLWH with major depression (MDD)..." is not clear and needs revision

- The paragraph now made clearer; see line number 406-410.

**Comments from reviewers # 2**

1. The authors tested neurologic performance and partially describe a representative cohort from their HIV care system. Their techniques use the combination of two screening tests and an ADL assessment to categorize the subjects with regard to HAND.

- I believe this is the background comment that the reviewer did not expect any response

2. There is no control population without HIV, and the association of the deficit with HIV is inferential.

- The author acknowledges that being a cross sectional study, the associations made are inferential at best and this is also explained as limitation in the discussion. Having a control population group would make this a case-control study which is beyond the scope.

3. The status of medical issues, cardiovascular risk and the like which increasingly are associated with poorer performance of HIV populations is in completely reported.

- The author agrees with the reviewers that cardiovascular and comorbidities on neurocognitive disorders have an interaction; however, since patients with cardiometabolic or cardiovascular disorders were excluded, the discussion of these factors on HAND was beyond the means and scope of the study.

4. The population is of interest since they have access to more effective therapy than many African populations previously studied. However, the interpretation of the findings is impossible in a cross sectional and uncontrolled use of tests not designed to achieve a secure diagnosis of HAND.

- The reviewer brings an important limitation as far as cross-sectional study is concerned; however, the author did not make any attempt to interpret findings beyond or make any causal relationship between dependent variable and explanatory. Furthermore, while the reviewer cited the limitation of the tests used, the author used two different tools to improve the sensitivity in the diagnosis of HAND. In settings where comprehensive neuropsychological performance cannot be done, MoCA and IHDS are the recommended instruments. The combined use of these tools has also been used elsewhere in Africa; see the study” 10.1186/s12883-020-01857-3” to improve the sensitivity of assessing HAND.

5. Associations with poor performance include advancing age and females are likely much over-interpreted in the discussion. Protective factors of education and compliance with treatment are expected. While the project represents a great effort, the manuscript attempts to over interpret it.

- I am not sure what reviewer’s suggestions are, given there are no questions or suggestions posed. While the reviewers considered the discussion was over interpreted, it should be noted that the author did not make any attempt to show any causal relationship but rather an effort to describe some of the plausible explanation that could be linked to our findings. Although, this is a research article from a cross-sectional study, a thorough literature review is necessary to explore the topic in detail and develop new related hypothesis. While the protective effect of education and ART adherence is expected, describing how this may happen is necessary, also the association between advanced age and female gender also need to be explored in detail.

6. Given emerging association cardiovascular risk factors, and co-morbidities, more emphasis on analysis of these may be also helpful.

- The author agrees with the reviewers that cardiovascular and comorbidities on neurocognitive disorders; however, since patients with cardiometabolic or cardiovascular disorders were excluded, the discussion of these factors on HAND was beyond the means and scope of the study.

7. Table 4 is hard to follow.

- Table is now made easier to follow.
